# Optimization of dye solutions for detecting damaged pancreatic tissues during islet isolation procedures

**DOI:** 10.1371/journal.pone.0255733

**Published:** 2021-08-13

**Authors:** Takehiro Imura, Akiko Inagaki, Yasuhiro Igarashi, Masafumi Goto

**Affiliations:** 1 Division of Transplantation and Regenerative Medicine, Tohoku University School of Medicine, Sendai, Japan; 2 Department of Surgery, Tohoku University Graduate School of Medicine, Sendai, Japan; University of Oklahoma Health Sciences Center, UNITED STATES

## Abstract

We previously reported that dye was effective to prevent the leakage of enzyme solutions from pancreatic glands during an islet isolation procedure. However, the dye used for islet isolation has not yet been optimized. In this study, we focused on pyoktanin blue (PB), diagnogreen (DG), and indigo carmine (IC) as potential candidates among clinically established dyes. A serial dilution assay was performed to determine minimal effective concentrations of each dye for detecting damaged pancreatic tissues. According to the outcome of serial dilution assays, double minimum effective concentrations of each dye were used for *in vitro* toxicity assays on islets and used in the isolation procedure to investigate whether they adversely affect islet isolation efficiency. The evaluations included islet yield, ADP/ATP, ATP/DNA, glucose stimulation test, and insulin/DNA assays. Islet viability cultured with PB contained medium was significantly lower than the other dyes. DG and IC appeared to be non-toxic to the islets. In isolation experiments, the islet yield in the DG group was considerably lower than that in the Control group, suggesting that DG might inhibit enzyme activity. The present study demonstrates that IC could be a promising candidate for an effective dye to detect damaged pancreatic tissues without affecting the enzyme activity and islet quality.

## Introduction

Pancreatic islet transplantation is an attractive and promising treatment for type 1 diabetic patients. However, many issues must be solved before islet transplantation is recognized as a standard therapy. One of the main weaknesses is the low success rate of islet isolation primarily based on insufficient islet yields [1, 2]. Although a low yield of isolated islets is caused by various factors, inadequate delivery of dissociation enzymes to the pancreatic parenchyma due to tissue damages during organ harvesting procedures could be a primary issue [3, 4].

We previously reported that both dye and glue were effective to prevent the leakage of enzyme solutions from pancreatic glands [3]. These methods are especially useful for the cases of islet autotransplantation associated with severe inflammation caused by pancreatic arteriovenous malformation [5, 6]. In our previous study, methylthionine chloride was proposed as a potential dye solution to detect the damaged area of the pancreas [3]. Methylthionine chloride was effective for this purpose but has been injected into humans only in limited cases, such as methemoglobinemia [7], because it was reported that methylthionine chloride may cause side effects such as nausea, vomiting, diarrhea, abdominal pain, cyanosis, and hemolytic anemia according to the applied dose [8]. Moreover, it is not readily available in some countries. Therefore, in terms of both regulation and availability, optimization of dye solutions for detecting damaged pancreatic tissues is needed.

In the present study, we focused on pyoktanin blue (PB), diagnogreen (DG), and indigo carmine (IC) as potential candidates among clinically established and readily available dyes. Optimization of these dye solutions was performed by serial dilution staining for pancreatic tissues, toxicity assays on isolated islets, and isolation experiments with dye-containing enzymes.

## Materials and methods

### Animals

Porcine pancreases were obtained at a local slaughterhouse from 2- to 4-year-old adult sows weighing 200 to 300 kg. Rat islets were isolated from inbred male Lewis rats (Japan SLC Inc., Shizuoka, Japan) weighing 288 to 306 g. All animals in this study were handled in accordance with the Guide for the Care and Use of Laboratory Animals published by the National Institutes of Health (Bethesda, MD, USA) [9] and the guidelines for animal experiment and related activities at Tohoku University except slaughterhouse pigs. The experimental protocol of the present study (protocol ID: 2016 Medical-Animal-197) was approved by the animal experimental committee in the Tohoku University. All surgical procedures for rats were performed under inhalation anesthesia using isoflurane, and every effort was made to reduce suffering. All rats were sacrificed with deep anesthesia and bleeding caused by cutting inferior vena cava.

### Dye chemicals

The following dyes were used in this study: PB (Kishida Chemical Co., Ltd., Osaka, Japan), DG (DAIICHI SANKYO Co., Ltd., Tokyo, Japan), and IC (DAIICHI SANKYO) (Table 1). These dyes are in widespread clinical use and readily available.

**Table 1:** Characteristics of dye solutions used in the study.

Trade Name	Component	Use application	Configuration	Color
**Pyoktanin Blue (PB)**	Methylrosanilinium chloride	Colon fiberscopy	Liquid	Violet
**Diagnogreen (DG)**	Indocyanine green	Liver function test	Powder	Green
**Indigo carmine (IC)**	Indigo carmine	Kidney function test	Liquid	Blue

### Dye concentrations

Porcine pancreases were obtained at a local slaughterhouse and immediately preserved in cold extracellular-type trehalose-containing Kyoto (ET-K) solution (Otsuka Pharmaceutical Factory Inc., Tokushima, Japan). In this study we used uncus of pancreas weighing 60 to 100g. Then, we inserted a cannula into the main pancreatic duct and cut the opposite site of the pancreatic parenchyma. A serially diluted concentration of each dye was injected into the pancreatic duct using a syringe through the cannula to determine the minimum effective concentrations for detecting the damaged area of pancreatic tissues. Dye concentrations in the pancreatic tissues were calculated by diluting the original dye concentration 15-fold, since 10 ml of dye solutions are typically diluted by 150 ml of dissociation enzyme solutions in clinical islet isolation. According to the outcome of serial dilution assays, double minimum effective concentrations of each dye were applied in the experimental studies to obtain the safety margin.

### Islet isolation and culture

Rat islet isolation was performed as described previously [10]. In brief, before the removal of the pancreas, the cannulated bile duct was injected with 10 mL of cold Hank’s balanced salt solution (HBSS) (Thermo Fisher Scientific Inc., Waltham, MA, USA) or dye solutions containing 1 mg/ml collagenase Type V (Sigma-Aldrich, St. Louis, MO, USA). After digestion at 37°C for 14 min, density-gradient centrifugation was performed using Histopaque-1119 (Sigma-Aldrich) and Lymphoprep™ (Nycomed Pharma AS, Oslo, Norway) to isolate the pancreatic islets. The islet count was performed as islet equivalents (IEQs) under a scaled microscope using diphenylthiocarbazone staining (Wako Pure Chemical Industries, Tokyo, Japan). The islets were cultured in Roswell Park Memorial Institute (RPMI) 1640 medium containing 5.5 mmol/L glucose (Thermo Fisher Scientific Inc.) and 10% fetal bovine serum (Equitech-Bio, Inc., Kerrville, TX, USA) at 37°C in 5% CO_2_ and humidified air.

### Islet viability and function

The adenosine diphosphate (ADP)/adenosine triphosphate (ATP) ratio assay [11, 12], ATP/deoxyribonucleic acid (DNA) assay [10, 13], static glucose stimulation test [14] in which the stimulation index was calculated by the amount of insulin in high glucose solution divided by the amount of insulin in low glucose solution, and insulin/DNA assay [14, 15] were performed as described previously.

### Statistical analysis

All results are reported as the means ± standard deviation (SD). One-way ANOVA followed by the Tukey test was used for the statistical evaluations. A P value < 0.05 was considered to be significant.

## Results

### Determination of the minimum effective concentrations of each dye for detecting damaged areas of pancreatic tissues

**Fig 1:**
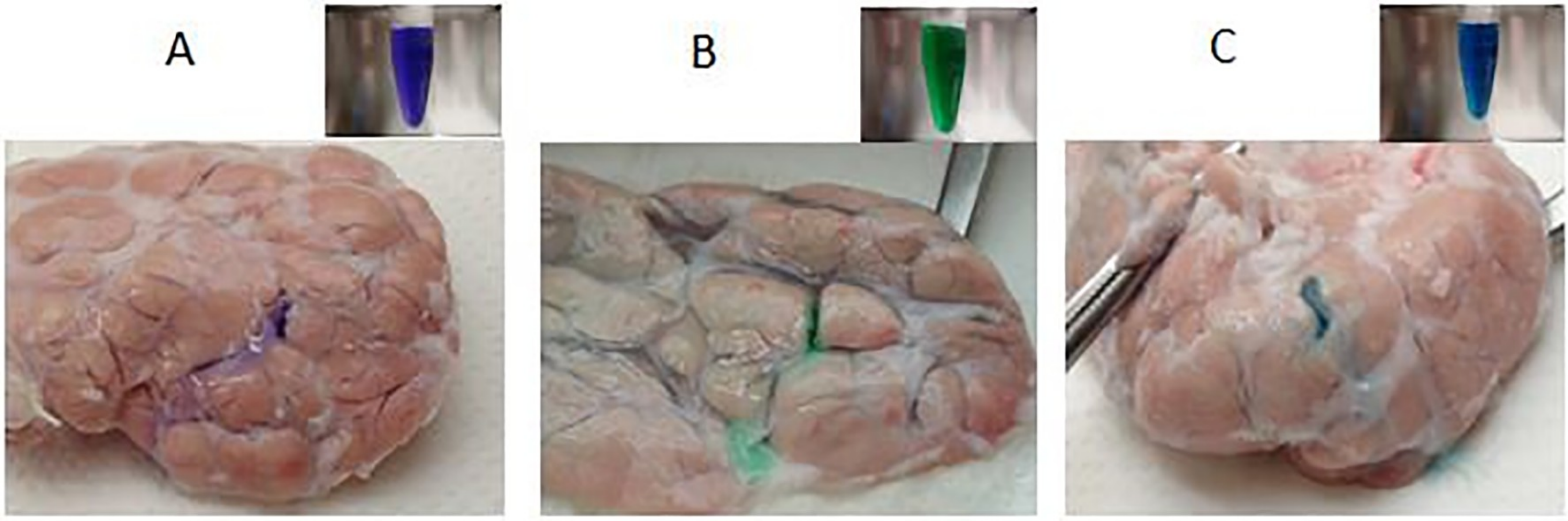
Determination of the minimum effective concentrations of each dye for detecting damaged areas of pancreatic tissues. A serial dilution assay revealed that the minimum effective concentrations of each dye for detecting damaged pancreatic tissues were 2.1 μg/ml (A: PB), 27.8 μg/ml (B: DG), and 6.7 μg/ml (C: IC), respectively.

A serial dilution assay revealed that the minimum effective concentrations of each dye for detecting damaged pancreatic tissues were 2.1 μg/ml (PB), 27.8 μg/ml (DG), and 6.7 μg/ml (IC), respectively (Fig 1). Therefore, in the following experiments aimed to investigate toxicity and adverse effects on isolation efficiency of each dye, the double minimum effective concentrations were applied in order to strictly assess the safety of each dye (PB: 4.2 μg/ml, DG: 55.6 μg/ml, and IC: 13.4 μg/ml).

### Toxicity of each dye to rat islets

**Fig 2:**
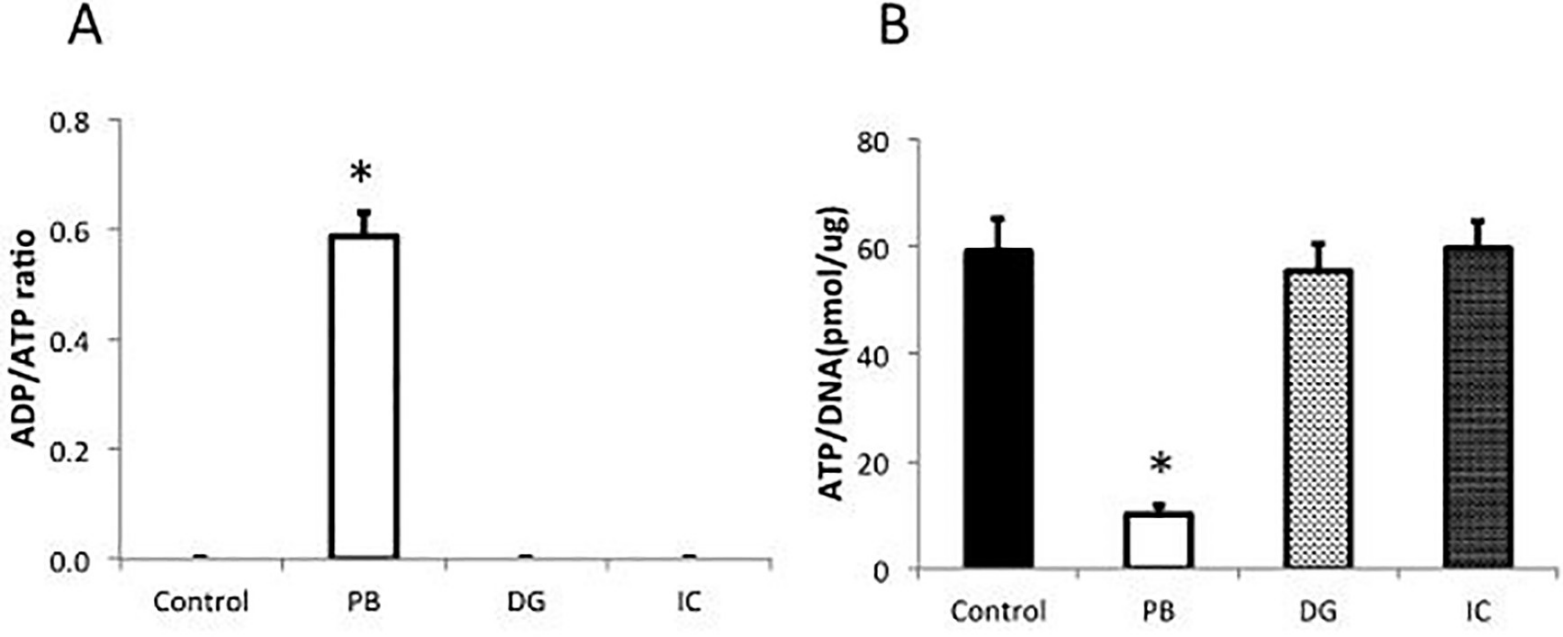
Toxicity of each dye to rat islets. **(A)** ADP/ATP ratio: The ADP/ATP ratio was significantly higher in the PB group than the other groups (n = 5, *p<0.05). **(B)** ATP/DNA ratio: The ATP/DNA ratio was significantly lower in the PB group than the other groups (n = 6, *p<0.05).

The isolated rat pancreatic islets were cultured for 18 hours in a culture medium containing each dye, and the toxicity of dye to the islets was evaluated by examining islet viability. The group without dye served as the control group. The ADP/ATP ratio was significantly higher in the PB group than the other groups (Control: 0.00, PB: 0.59±0.04, DG: 0.00, and IC: 0.00, n = 5, p<0.05) (Fig 2A). On the other hand, the ATP/DNA ratio was significantly lower in the PB group than the other groups (Control: 59.1±6.0, PB: 10.1±1.5, DG: 55.3±5.1, and IC: 59.7±5.3 pmol/μg, n = 6, p<0.05) (Fig 2B).

### Influence of each dye on the yield of isolated rat islets

According to the outcome of the above toxicity assay, we focused on DG and IC in the following experiments aimed to examine whether islet yield is affected by each dye. Islet isolation was performed using an enzyme solution containing each dye. The group without dye served as the control group.

**Fig 3:**
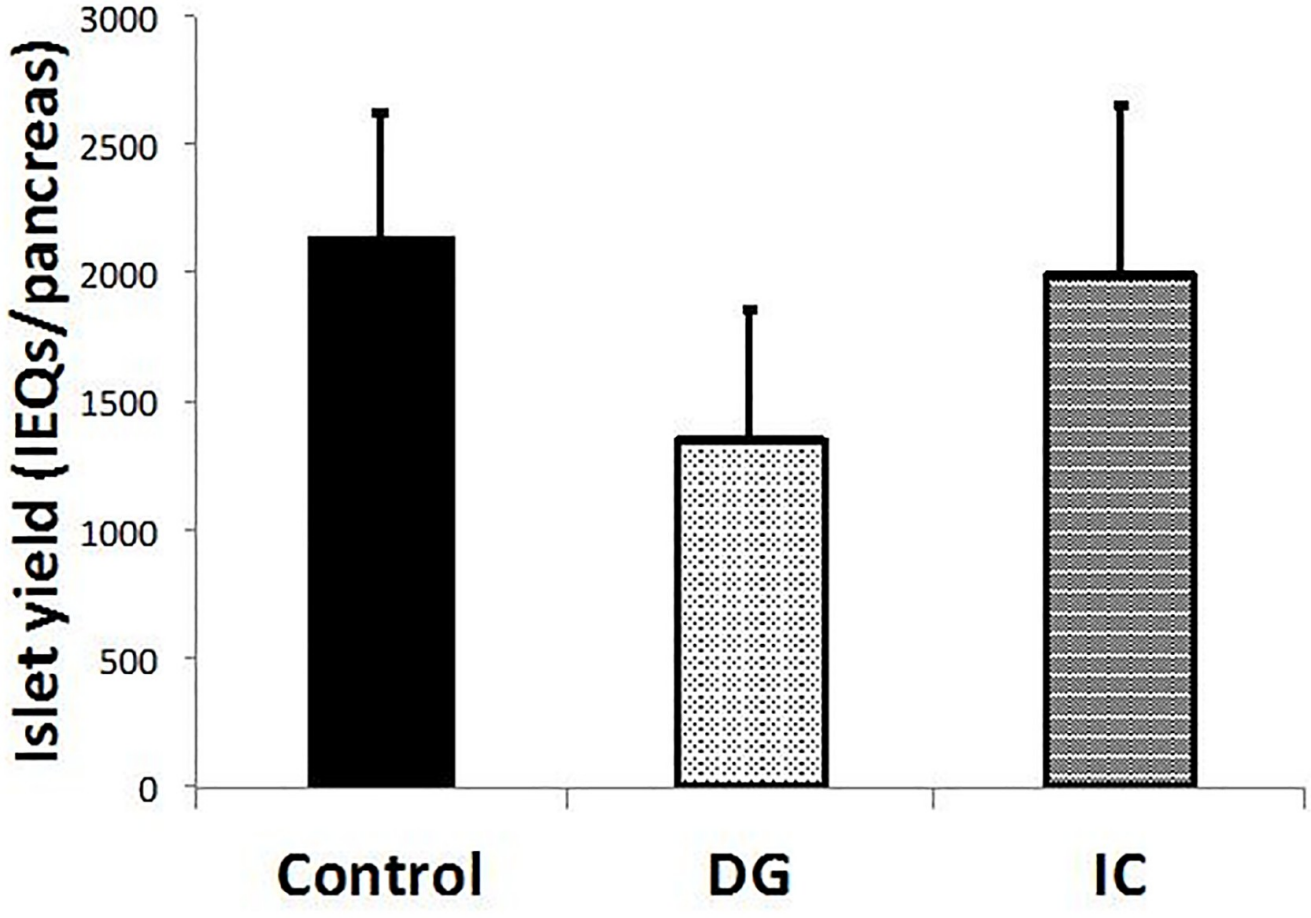
Influence of each dye on the yield of isolated rat islets. The islet yield after purification was considerably lower in the DG group compared with the Control and IC groups (n = 5).

The islet yield after purification was considerably lower in the DG group compared with the Control and IC groups (Control: 2,146 ± 476, DG: 1,348 ± 510, and IC: 1,990 ± 665 IEQs/pancreas, n = 5) (Fig 3). Notably, tiny precipitated particles were often observed in the DG group.

### Influence of each dye on the viability and function of isolated rat islets

**Fig 4:**
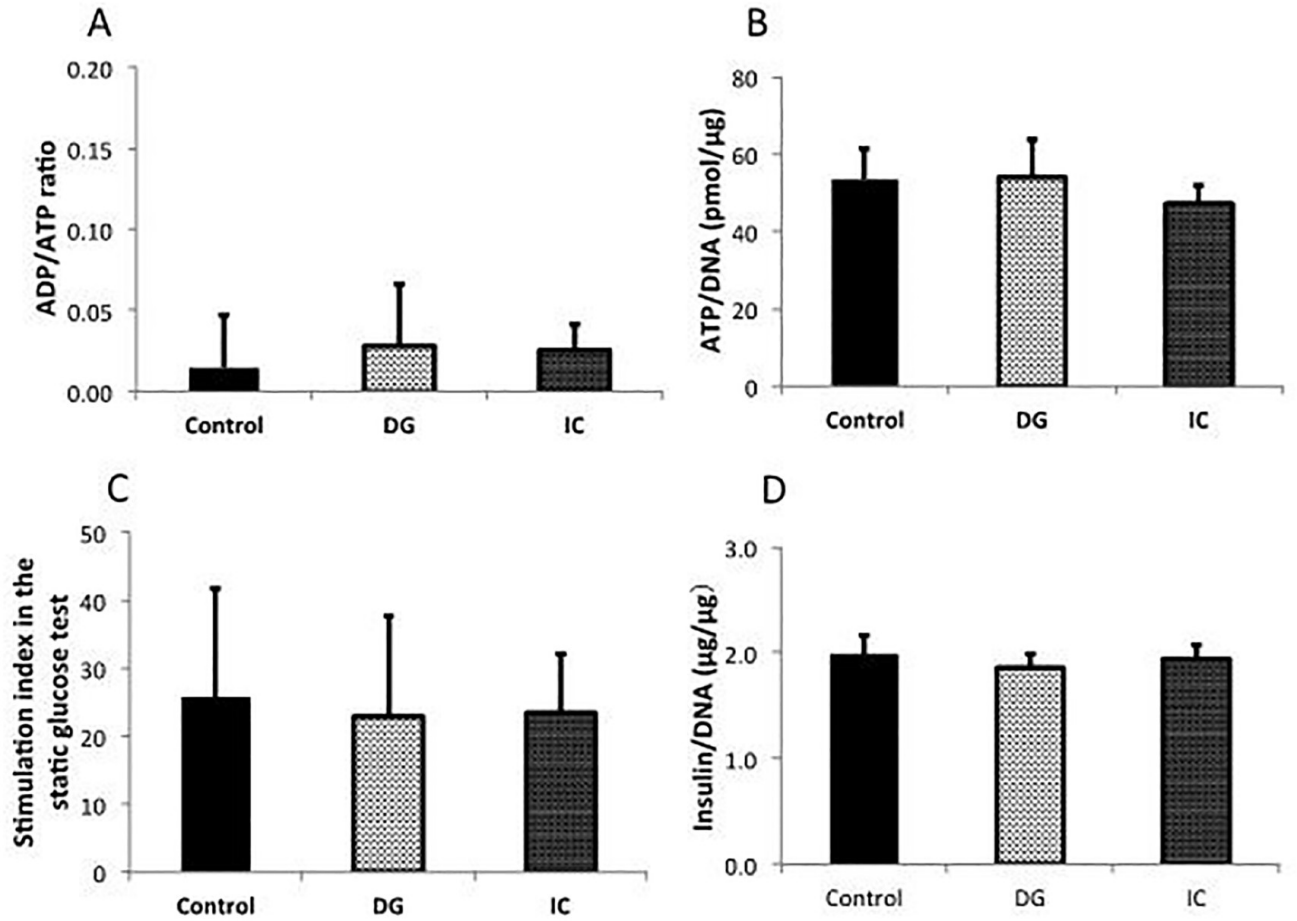
Influence of each dye on the viability and function of isolated rat islets. **(A)** ADP/ATP ratio, **(B)** ATP/DNA ratio, **(C)** stimulation index in the static glucose stimulation test, and **(D)** insulin/DNA ratio. No significant differences were observed among the groups.

The viability and function of isolated islets were evaluated using the islets obtained in the abovementioned experiment for evaluating the yield of islets. Regarding the islet viability and function, no significant differences were observed among the groups in the ADP/ATP ratio (Control: 0.01±0.03, DG: 0.03±0.04, and IC: 0.03±0.02, n = 5) (Fig 4A), the ATP/DNA ratio (Control: 53.4±8.1, DG: 54.0±10.0, and IC: 47.2±4.6 pmol/μg, n = 5) (Fig 4B), the stimulation index in the static glucose stimulation test (Control: 25.8±16.0, DG: 22.9±14.8, and IC: 23.4±8.7, n = 5) (Fig 4C), or the insulin/DNA ratio (Control: 1.98±0.43, DG: 1.86±0.30, and IC: 1.94±0.29 μg/μg, n = 5) (Fig 4D).

## Discussion

It was previously shown that dye is effective for detecting the damaged area of pancreatic tissues during islet isolation procedure [3]. In addition to identifying the damaged portion of the pancreases, dye can also identify the part where the enzyme has not reached due to the cases in which the accessory pancreatic duct is not connected to the main pancreatic duct or the pancreatic duct is clogged. In these cases, it is possible to increase the islet yield by directly re-injecting the enzyme into the unperfused area. However, no investigation has been performed to optimize the suitable dye for pancreatic islet isolation. In the present study, we only focused on optimizing the suitable dye for islet isolation, and compared the effectiveness and compatibility of three commonly used dyes (PB, DG, and IC) in the clinical setting with islet isolation. Consequently, IC was revealed to be the preferable dye for islet isolation.

In this study, PB, DG, and IC were selected as potential candidate dyes among suitable dyes for islet isolation, because all of them have been widely used in the clinical field. For instance, PB is commonly used for the pit pattern diagnosis in colon fiberscopy [16], and DG is typically applied in the liver function assay [17] (Table 1). In addition, IC is frequently utilized for renal function testing and sentinel lymph node mapping [18] (Table 1). Therefore, all of these chemicals are speculated to have a rather low risk of causing side effects in transplant recipients.

A serial dilution assay to identify the minimum effective concentrations of each dye clearly showed that the tinting strength per unit was the highest with PB and the lowest with DG. Despite the fact that the required amount of PB was extremely lower than other chemicals, the viability of cultured islets was significantly lower in the PB group compared with the other groups. These data demonstrated that PB is detrimental for the islets for an unidentified reason and is not suitable for islet isolation.

Regarding the toxicity of DG and IC on the islets, the isolated islets maintained good viability despite being cultured for 18 hours in a medium containing twice the clinical level of dye. Given that the exposure time of the islets to dye solutions is at longest one hour in the clinical setting [3, 19], one can conclude that DG and IC are not toxic to the islets.

Of particular note, in islet isolation experiments which were only performed to evaluate the toxicity of each dye to the islets, the islet yield in the DG group was considerably lower than that in the Control group, whereas the islet yield in the IC group was comparable with that in the Control group. However, both the viability and function of the isolated islets were well maintained even in the DG group. Furthermore, tiny precipitated particles were often observed in the DG group. All these data suggested that DG might inhibit the dissociation enzyme activity, likely due to oligomer formation with calcium ion [20].

Concerning the physical property, DG must be dissolved in an attached solution since it is a powder. However, IC is available as a liquid, therefore it is easy to handle and is expected to have a lower risk of contamination. Taken together, IC is considered to be easy to handle, non-toxic to the islets, have a good isolation efficiency and low risk of contamination, and is readily available.

In conclusion, the present study demonstrated that IC is a promising and effective dye to detect damaged pancreatic tissues without affecting the islet isolation efficiency or the quality of isolated islets.
